# Improved genome assembly of whale shark, the world’s biggest fish: revealing intragenomic heterogeneity in molecular evolution

**DOI:** 10.1093/gigascience/giag014

**Published:** 2026-02-06

**Authors:** Yawako W Kawaguchi, Rui Matsumoto, Shigehiro Kuraku

**Affiliations:** Molecular Life History Laboratory, Department of Genomics and Evolutionary Biology, National Institute of Genetics, Yata 1111, Mishima, Shizuoka 411-8540, Japan; Okinawa Churashima Research Center, Okinawa Churashima Foundation, Aza Ishikawa 888, Motobu, Kunigami, Okinawa 905-0206, Japan; Okinawa Churaumi Aquarium, Ishikawa 424, Motobu, Kunigami, Okinawa 905-0206, Japan; Molecular Life History Laboratory, Department of Genomics and Evolutionary Biology, National Institute of Genetics, Yata 1111, Mishima, Shizuoka 411-8540, Japan; Department of Genetics, Sokendai (Graduate University for Advanced Studies), Yata 1111, Mishima, Shizuoka 411-8540, Japan; Laboratory for Phyloinformatics, RIKEN Center for Biosystems Dynamics Research, Minatojima-minamimachi 2-2-3, Chuo, Kobe, Hyogo 657-0024, Japan

**Keywords:** whale shark, genome assembly, sex chromosome, male-driven evolution, intragenomic heterogeneity

## Abstract

**Background:**

High-quality chromosome-level assemblies are essential for understanding genome evolution but remain difficult to obtain for complex genomes such as those of vertebrates. The whale shark (*Rhincodon typus*) is an endangered species whose ecology and evolutionary history remain poorly understood. Although genomic resources have been developed for this species, available assemblies have left key uncertainties in the chromosome structure.

**Results:**

We generated a near gap-free genome assembly of the whale shark using long-read sequencing and Hi-C scaffolding, markedly improving contiguity and completeness. In particular, the X chromosome was extended to nearly twice its previous length, and putative pseudoautosomal regions were identified. Moreover, we report the first Y-linked scaffolds for this species. Comparative analyses with the zebra shark revealed exceptionally low substitution rates across the genome. We further detected a negative correlation between chromosome length and synonymous substitution rate (*d_S_*), explained by a positional gradient, here referred to as “chromocline,” in which substitution rates gradually decrease from chromosomal ends toward central regions. Notably, the X chromosome exhibited lower *d_S_* compared with autosomes of similar size, consistent with male-driven evolution.

**Conclusions:**

This improved whale shark genome assembly overcomes major limitations of previous resources and enables refined analyses of sex chromosomes and genome-wide evolutionary dynamics. The enhanced sex chromosome resources provide a foundation for deeper investigations of reproductive biology in this species. Increased contiguity also reveals pronounced intragenomic heterogeneity in molecular evolutionary rates, indicating that positional effects and sex chromosome differences are key determinants of synonymous divergence. This resource will facilitate population genetic and conservation genomic studies in the whale shark.

## Background

Achieving complete and accurate genome assemblies remains a significant challenge in genome informatics. It is hindered primarily by intrinsic genomic complexities such as large size, high repetitiveness, and heterozygosity, compounded by limitations in sequencing data quality and quantity. Technological advancements and accumulated experience are making complete genome sequencing more accessible [1], but in reality, genome assembly is often an iterative process, leading to multiple versions for a single species, frequently generated by diverse research entities. Existing guidelines largely focus on initial assembly finalization [2], often neglecting critical considerations for releasing improved versions to avoid traceability issues, particularly regarding consistency of chromosome and sequence identifiers. This oversight severely impedes data interoperability, reusability, and reproducibility. Thus, recommended practices for iterative genome assembly improvement and the subsequent formal release of updated versions remain to be formulated.

The species chosen in this study, whale shark, *Rhincodon typus*, is the largest “fish” species among extant fish lineages encompassing jawless, chondrichthyan, and osteichthyan species. The whale shark is categorized as endangered (EN) in the IUCN Red List, which limits tissue sampling for biological studies. Genome sequencing for this species was initiated with assembling short reads by Read et al. to obtain highly fragmented contigs [3]. This study used a postmortem male tissue sample at the Georgia Aquarium. Later, Hara et al. performed reassembly of the short reads obtained by Read et al. [4], and scaffolded the contigs with mate pair reads produced using blood cell DNA with mate-pair library preparation protocol iMate [5]. In parallel, Weber et al. obtained heart tissue from a deceased male at Hanwha Aquarium, Jeju, Korea, and combined short reads and mate-pair, with TruSeq Synthetic Long Read libraries to obtain the assembly RhiTyp_1.0 [6]. In 2021, the residual tissue sample used by Read et al. was used for obtaining consensus long reads with single-molecule, real-time technology of Pacific Biosciences [7]. The first release of chromosome-scale assemblies was achieved by DNA Zoo consortium, which scaffolded the above-mentioned assembly sequences by Weber et al. with Hi-C data prepared with male tissues [8]. Most recently, Yamaguchi et al. utilized the 10X Chromium Linked-Read data [9] as part of data production in the Squalomix consortium [10]. The contigs resulted from this data were scaffolded by blood cell Hi-C data prepared with the iconHi-C protocol. This assembly, sRhiTyp1.1, is derived from the sexually mature male individual (named Jinta) maintained at Okinawa Churaumi Aquarium since 1995 [11] and is labeled as “reference” at NCBI Genomes, as of October 2025.

Recent advances have enabled the identification of sex chromosome sequences in multiple cartilaginous fishes, a group known to exhibit male heterogamety [12]. While contiguous X chromosomes have been reported for some species, their completeness and taxonomic coverage remain limited [9, 13–15]. Y chromosomes, often highly repetitive and small, are even more elusive; only a few reports have constructed partial sequences identified to date [13–15]. Notably, sex chromosomes of sharks and rays are thought to have been conserved for over 300 million years, underscoring the evolutionary importance of expanding genomic insights into these chromosomes [14]. In whale shark, an X chromosome was previously identified by Yamaguchi et al. [9], but this effort resulted in relatively short (∼12 Mbp) and fragmentary scaffold, compared with the ∼20 Mbp-long X chromosome of its close relative, the zebra shark. No sequences from the Y chromosome have been reported for this species [9]. Thus, more complete X chromosome assemblies and the discovery of Y-linked sequences across broader taxa are essential to understand sex chromosome evolution in this lineage.

Resolving chromosome-scale assemblies, including the sex chromosomes, provides a window into how genomic position shapes evolutionary rates. Gene evolutionary rates are not solely determined by gene function but are also influenced by their genomic location [16–18]. For example, in the sex chromosome context, male-driven evolution predicts lower neutral substitution on the X chromosome than on autosomes [19–21], whereas hemizygosity can accelerate adaptive change on X chromosomes (faster-X effect [22]). Beyond sex linkage, both birds and mammals have shown faster evolution on shorter chromosomes [23–25]. One theoretically grounded explanation is that the obligatory crossover elevates recombination per unit length on small chromosomes, and recombination-associated break/repair together with linked selection or GC-biased gene conversion can raise local substitution rates [17, 26]. Positional effects also occur within chromosomes; in *Xenopus*, synonymous divergence increases with distance from centromeres and tends to be higher toward chromosomal ends [27]. These patterns collectively motivate joint tests of genomic location, chromosome size, and sex linkage. However, strong intrachromosomal heterogeneity in recombination further complicates inference, and comprehensive analyses that disentangle these factors remain scarce outside Tetrapoda.

In this study, we present a new chromosome-level genome assembly for the whale shark. Our assembly reveals a more complete X chromosome and, for the first time, putative Y chromosome sequences. Leveraging this new resource, we test the influence of chromosome length, chromosomal position, and sex linkage on synonymous substitution rates. These analyses reveal positional and sex-chromosome effects on molecular evolutionary rates, providing a foundation for future comparative studies.

## Results

### Long-read-based chromosome-scale genome assembly of whale shark

**Figure 1:**
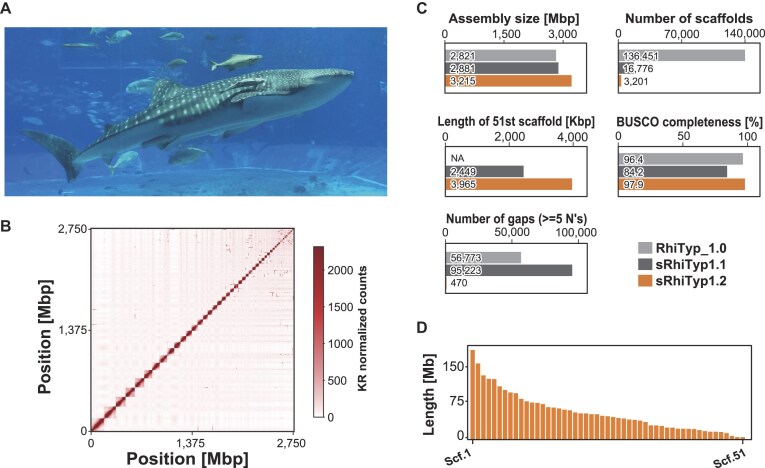
Statistics of the new genome assembly of whale shark (*Rhincodon typus*). (A) Whale shark individual, named Jinta, from which a genome assembly was obtained in this study. Photo credit: Akifumi Yao. (B) Hi-C contact map of top 51 scaffolds, showing chromatin contact profiles supporting chromosome-scale scaffolding. (C) Comparative assembly statistics between the previous versions (RhiTyp_1.0 and sRhiTyp1.1) and our new version (sRhiTyp1.2). (D) Length distribution of the 51 longest scaffolds, defined as the chromosomes. Abbreviations: Scf, scaffold; Chr, chromosome.

We generated a new genome assembly for the whale shark (*Rhincodon typus*) by conducting long-read sequencing of an adult male individual, followed by *de novo* assembly with Hi-C scaffolding (Fig. 1A, B). Our new assembly (named sRhiTyp1.2) has a total size of 3.22 Gbp and consists of 3,201 scaffolds. Compared to 2 previous versions (RhiTyp_1.0 and sRhiTyp1.1), which were 2.82 Gbp in size with 136,451 scaffolds and 2.88 Gbp in size with 16,776 scaffolds, respectively, the new assembly exhibits a marked improvement in contiguity (Fig. 1C and Supplementary Table S1) [6, 9]. Furthermore, the assembly size of sRhiTyp1.2 now approximates the estimated genome size of 3.75 Gbp previously measured with flow cytometry [1]. Notably, compared to sRhiTyp1.1, the number of gaps decreased remarkably (95,223 to 470), and the BUSCO completeness score strikingly increased (84.2 to 97.9%, Fig. 1C and Supplementary Table S1). These enhancements indicate a more complete chromosome-level assembly.

We defined the putative chromosome set by selecting the 51 largest scaffolds (Fig. 1D), based on the known chromosome number of the whale shark [12], and designated them as chromosome-scale sequences for downstream analyses. Within this set, the smallest chromosome (scaffold 51) was extended from 2.45 to 3.97 Mbp compared to sRhiTyp1.1. The chromosome lengths vary from 3.97 to 185 Mbp, which reflects a typical length spectrum observed in cartilaginous fishes [9, 28].

### More complete sex chromosome sequencing

**Figure 2:**
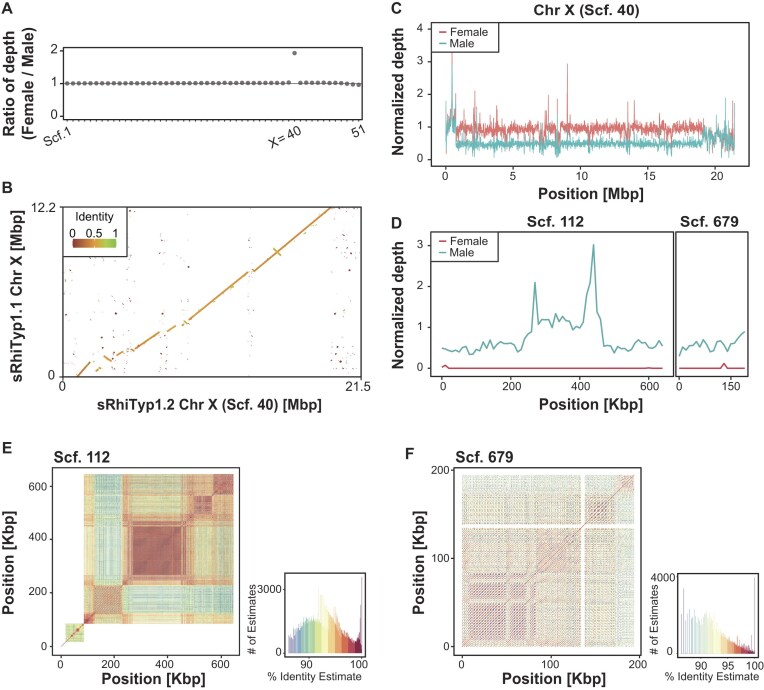
Identification and characterization of sex chromosomes. (A) Female-to-male ratio and median of the depth of genome short reads for each chromosome. Scaffold 40 displays approximately twice the read depth in females compared to males, consistent with an X chromosome. (B) Dot plot of the X chromosome comparing the new assembly with the previous assembly (sRhiTyp1.1). (C) Depth distribution in the X chromosome. (D) Depth distribution in the 2 putative Y chromosome fragments. (E, F) Identity heatmaps for the 2 putative Y chromosome fragments. Abbreviation: Scf, scaffold.

To identify sex chromosomes, we mapped short-read whole-genome sequencing data from both male and female individuals to the new assembly. Among the 51 scaffolds presumed to represent chromosomes, scaffold 40 exhibited a male-to-female read depth ratio of ~1:2, a hallmark of the X chromosome in a species of male heterogamety. Based on this pattern, we designated it as the X chromosome (Fig. 2A). The X chromosome in the new assembly was nearly twice as long as in the previous version (increased from 12 to 21 Mb; Fig 2B), and the number of annotated genes increased from 247 to 281. Regions at both ends of the X chromosome exhibited similar depth between sexes (Fig. 2C), suggesting the presence of pseudoautosomal regions (PARs), in which recombination may occur between X and Y chromosomes.

We further searched for Y-linked scaffolds by identifying regions with high read depth in males but low or no coverage in females. This approach led to the identification of 2 scaffolds with the differential mapping results between the sexes that are presumed to be fragments of the Y chromosome: scaffold 112 (645,398 bp) and scaffold 679 (195,000 bp) (Fig. 2D). Each of these 2 scaffolds contained only a few predicted genes (2 and 1, respectively), and none of those genes were found to be implicated in sex determination documented in other species so far. Because scaffolds with male-restricted coverage could in principle result from contaminants specific to the sequenced male individual, we verified their origin by BLAST searches against the NCBI nr database. The predicted genes exhibited strong matches to homologs from other elasmobranch species, demonstrating that these scaffolds represent genuine shark sequences rather than contaminants. Their sequence composition was characterized by a high abundance of repeats (Fig. 2E, F). This repeat-rich and gene-poor pattern is consistent with previous observations of Y chromosomes in other shark species [14].

### Substitution rate variation within and between chromosomes

**Figure 3:**
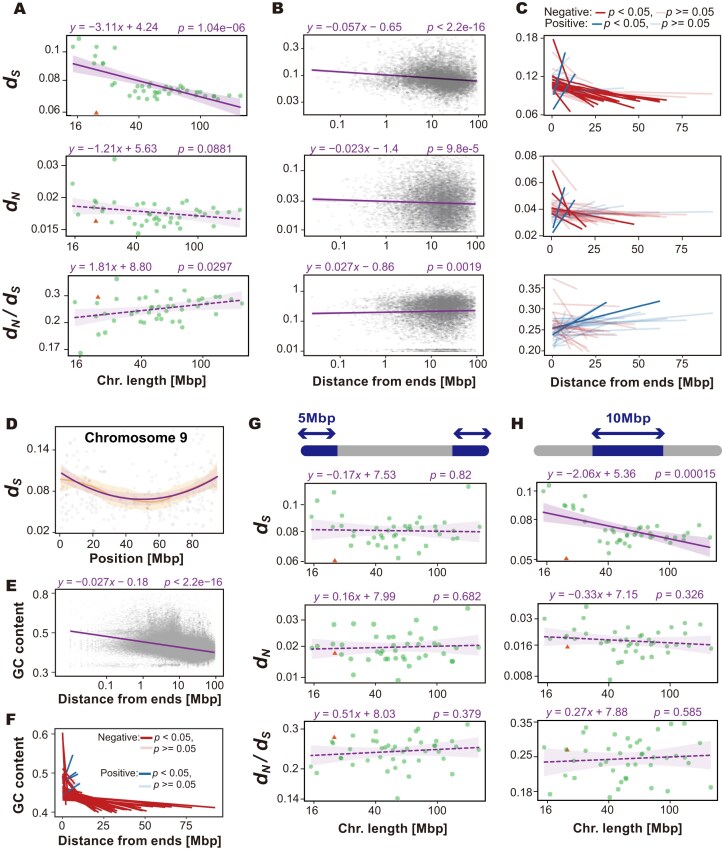
Substitution rates and GC contents across chromosomal regions. (A) Chromosome lengths plotted against the median substitution rates for chromosomes containing more than 10 genes. (B) Substitution rates of individual genes plotted against their distance from chromosome ends. (C) Slope estimates from regressions between substitution rates and distance from chromosome ends, calculated separately for each chromosome. (D) *d_S_* distribution within chromosome 9. Puple line and area show quadratic regression result and 95% confidence, respectively. Orange line and area are LOESS regression results and 95% confidence, respectively. The regression results and the plots of other chromosomes are shown in Supplementary Figs S1 and S2. (E) GC content in 10-kbp windows plotted against their distance from chromosome ends. (F) Slope estimates from regressions between GC content and distance from chromosome ends, calculated for individual chromosomes. (G) Chromosome lengths plotted against the median substitution rates, for only genes located within 5 Mbp of chromosome ends. (H) Chromosome lengths plotted against the median substitution rates, for only genes located within 10 Mbp-long stretches at the centers of chromosomes. In (A), (G), and (H), circles and triangles indicate autosomes and the X chromosome, respectively. Solid and dashed lines indicate significant (*P* < 0.05) and non-significant simple regressions, respectively; shaded areas represent 95% confidence intervals. All axes in (A), (B), (E), (G), and (H) are shown on logarithmic scales. Abbreviation: Chr, chromosome.

To assess regional patterns of molecular evolution, we quantified substitution rates by comparing orthologous protein-coding genes between the whale shark and the zebra shark (*Stegostoma tigrinum*), the closest extant relative of the former species. Because some chromosomes harbor only a few orthologs, which can yield unstable median estimates, we summarized per-chromosome rates only for chromosomes containing more than 10 orthologs. Across these chromosomes, substitution rates varied appreciably: the median values of synonymous substitution (*d_S_*) ranged from 0.0612 to 0.111, non-synonymous substitution (*d_N_*) from 0.0153 to 0.0339, and *d_N_*/*d_S_* from 0.173 to 0.326 (Fig. 3A). Notably, *d_S_* showed a significant negative correlation with chromosome length, while *d_N_* exhibited a weaker but still negative trend (Fig. 3A), pointing to an effect of chromosome length on substitution rates. Accordingly, shorter chromosomes show higher neutral substitution rates, with only a modest increase in non-synonymous rates.

To scrutinize this pattern, we analyzed the relationship between gene position (distance from chromosome ends) and substitution rate. We found that genes located closer to the ends tended to exhibit higher *d_S_* and *d_N_* significantly (Fig. 3B). At the individual chromosome level, 35 out of the 44 chromosomes showed a negative correlation between *d_S_* and distance from the chromosome ends, 19 of which were statistically significant, including the X chromosome (Fig. 3C, *P* < 0.05). In contrast, only 9 chromosomes showed a positive correlation, with only 2 of them showing significance.

To visualize a representative example of this intrachromosomal pattern, we plotted the positional distribution of *d_S_* along chromosome 9 (Fig. 3D). In this chromosome, substitution rates are clearly elevated near both ends and lower toward the center, forming a significant U-shaped regression pattern. Similar patterns were observed in most other chromosomes (chromosomes 1–20; Supplementary Figs S1 and S2), although not all individual correlations reached statistical significance. This general tendency toward end-biased, concave profiles further supports the presence of a consistent positional gradient in substitution rates across the genome.

GC content is also negatively correlated with distance from chromosome ends (Fig. 3E, F). At the individual chromosome level, most chromosomes also displayed negative correlations. Together, these patterns indicate pronounced intrachromosomal heterogeneity, manifesting as a positional cline with distance from chromosome ends.

The intrachromosomal heterogeneity could underlie the chromosome-length effect, because shorter chromosomes harbor a larger fraction of end-proximal, fast-evolving genes than larger chromosomes do. We therefore asked whether the length effect is independent of intrachromosomal heterogeneity, specifically whether it persists at a fixed intrachromosomal position. Focusing on only genes within 5 Mb of the ends renders the length effect undetectable (Fig. 3G). It indicates that the length-dependent variation in nucleotide substitution rates is largely driven by gene positioning along the chromosome. In contrast, focusing on only genes located in the central regions of chromosomes (within the central 10 Mbp), the length effect persisted (Fig. 3H). This suggests that the variations in substitution rates are better explained by proximity to chromosome ends than to central regions—supporting a model in which distance from the ends rather than from the center is the primary positional driver of intrachromosomal substitution rate variation.

**Figure 4:**
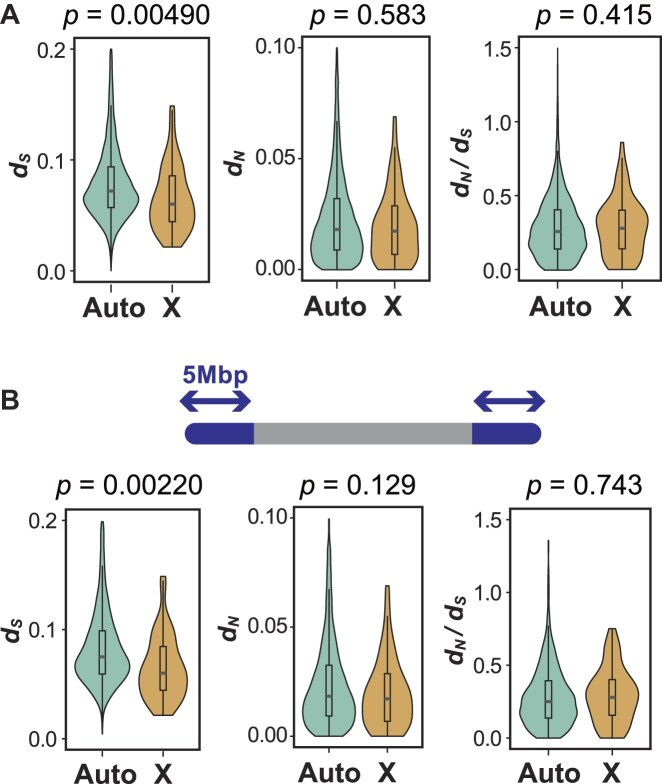
Chromosomal distribution of substitution rates. (A) Comparison of substitution rates between autosomes and the X chromosome using all genes. (B) Focusing on the only genes within 5 Mbp of chromosome ends. Bars and boxes represent the median and interquartile range, respectively; whiskers extend to 1.5 times the interquartile range. Statistical significance was assessed using a randomization test with 10,000 permutations. Abbreviations: Auto, autosome; X, X chromosome.

An exception to this overall pattern was observed in the X chromosome (scaffold 40). Although it is one of the relatively short chromosomes (40th out of 51), it exhibited significantly lower *d_S_* compared to autosomes (Figs 3A and 4A). *d_N_* was not significantly different, and *d_N_*/*d_S_* was slightly but not significantly elevated on the X chromosome. Notably, this reduction in *d_S_* remained significant even when only telomere-proximal genes were analyzed (Figs 3G and 4B), suggesting that the X chromosome has intrinsically lower substitution rates, independent of gene position. This pattern is consistent with a male-driven evolution in this species. To evaluate this more directly, we sought to estimate *d_S_* for the Y chromosome; however, the putative Y scaffolds contained too few genes to support reliable rate estimates and were therefore excluded.

## Discussion

This study provided a new genome assembly sRhiTyp1.2 of the whale shark. It also serves as a case study for a frequent issue in modern genomics: the release of a new genome assembly for a species that already has one. Crucially, its importance is infrequently emphasized in scientific publications. Considering the coherence between different versions, we adjusted the nucleotide sequence orientations of the individual chromosomes to those in the previous assembly sRhiTyp1.1. Importantly, this is essential for confirming the location of genes and other genomic elements and avoiding traceability issues. In fact, maintaining consistent chromosome identifiers is dependent on successfully assembling and identifying complete chromosomes. Consideration of these 2 items is demanded in finalizing the assembly sequence set to be released in public. However, successful retrieval of individual chromosomes, even for relatively small ones, is a prerequisite for maintaining the chromosome identifiers. In our present study, scaffold sequences in the novel assembly sRhiTyp1.2 were sorted by their lengths and given new identifiers according to their lengths. This is because some of the scaffolds in the previous assembly version were supposedly fragments of large chromosomes but not entire chromosomes. When complete sequences become available, further reorientation should be considered based on centromere positions to designate the end of individual short chromosome arms as the beginning of the chromosomal sequences.

Our new whale shark assembly substantially reduces gaps and extends the X chromosome, yielding far more complete chromosome resolution than prior versions (Figs 1C and 2B). Although previous efforts had achieved near chromosome-scale assemblies using short reads combined with Hi-C scaffolding, the contigs in those assemblies were relatively short, necessitating the insertion of large gaps during scaffolding [9]. In contrast, our approach utilized long-read sequencing with Oxford Nanopore technology, which dramatically reduced the number of gaps and improved assembly contiguity. This enabled more precise comparative analyses with the closely related zebra shark, whose genome was previously studied at the chromosome level. In addition, we identified, for the first time in this species, 2 putative scaffolds from the Y chromosome (Fig. 2D, E, and F). The gene repertoires on these scaffolds were extremely sparse, consistent with previous reports of the Y chromosomes of other shark species, and the high repeat content resembled that of the bamboo shark, a close relative [14]. Furthermore, the X chromosome in our assembly was nearly twice as long as the counterpart in the previous version, and we identified putative PAR at 2 ends, which were not detected in earlier assemblies.

Using this improved assembly, we quantified genome-wide synonymous divergence between whale shark and zebra shark, whose divergence is estimated at ~50 million years ago [29]. We found that the median synonymous substitution rates (*d_S_*) across chromosomes ranged from 0.0612 to 0.111, suggesting exceptionally slow rates of molecular evolution (Fig. 3A). For comparison, the human–mouse median *d_S_* is ~0.58 over 90 million years [30], the chicken–zebra finch *d_S_*is ~0.4 over 66–86.5 million years [31, 32], and the *Tetraodon*–*Takifugu d_S_* is ~0.59 over 32–55 million years [32, 33]; reviewed in [34]. These comparisons underscore the exceptionally slow molecular clock of these shark lineages.

**Figure 5:**
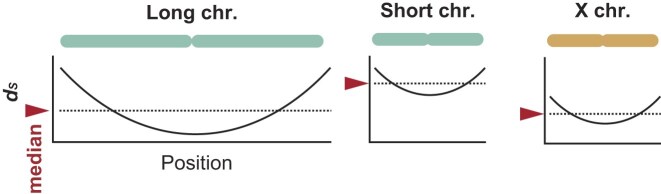
Schematic diagrams of the *d_S_* distribution along chromosomes, “chromocline.” Longer chromosomes have more genes located far from the ends, resulting in a lower overall median *d_S_* (arrows). Conversely, shorter chromosomes tend to have higher median *d_S_* because they have fewer genes located far from their ends. The X chromosome, however, exhibits unusually low median *d_S_* despite its small length. Abbreviation: Chr, chromosome.

Within this overall slow-evolving background, we observed a negative correlation between chromosome length and synonymous substitution rate (Fig. 3A), consistent with prior studies [23–25, 35]. We also detected pronounced intrachromosomal heterogeneity in substitution rates (Fig. 3B, C, D, G, and H). This intrachromosomal heterogeneity is consistent with previous reports that telomere-proximal (subtelomeric) regions often exhibit accelerated molecular evolution across diverse taxa [36–39]. In the whale shark genome, we further found that substitution rates change gradually as the distance from chromosome ends increases across many chromosomes (Figs. 3D and 5), forming a positional cline that we refer to here as “chromocline.”

Importantly, our results suggest that the negative correlation between chromosome length and chromosome-level summaries of *d_S_* does not reflect an independent property of chromosome size. When a single representative rate per chromosome (e.g., the median or mean across genes) is computed, it is inherently influenced by gene position (Fig. 5). Longer chromosomes contain a greater fraction of genes at larger absolute distances from chromosome ends, which lowers their chromosome-level summaries, whereas shorter chromosomes are relatively enriched for telomere-proximal genes with elevated rates. This compositional effect produces the apparent negative correlation between chromosome length and synonymous substitution rate. Consistent with a shared positional mechanism, GC content exhibits a parallel cline, decreasing with distance from chromosome ends (Fig. 3E, F). These coupled gradients in substitution rates and base composition indicate that chromosome-length effects on molecular evolution are not intrinsic properties of chromosome size per se, but instead reflect the spatial distribution of genes along chromosomes aligning “chromocline.” This result demonstrates that the chromosome length dependence of substitution rates is not an intrinsic property of chromosome size, but rather reflects the distribution of genes with respect to distance from the ends. Notably, a similar gradient of synonymous substitution rate has been reported in *Xenopus* [27], raising the possibility that the widely observed chromosome-length effect reflects a conserved “chromocline.”

Such elevated rates near telomeres may reflect increased recombination activity in subtelomeric regions—a pattern commonly reported in animals [40, 41]. Alternatively, or in addition, chromatin state and DNA repair mechanisms may differ between distal and central chromosomal regions, contributing to the observed variation [42, 43]. The consistent effect of this “chromocline” across many chromosomes in the whale shark genome provides an explanation for the inter-chromosomal variation in nucleotide substitution rates. A few chromosomes showed higher synonymous substitution rates toward their centers. This heterogeneity may reflect chromosome-specific landscapes in recombination or chromatin state. However, since these chromosomes were also among the shortest, we cannot exclude a possibility that they represent assembly fragments of larger chromosomes, so we interpret these slopes cautiously.

Interestingly, despite being one of the relatively short chromosomes (40th out of 51), the X chromosome exhibited significantly lower synonymous substitution values than autosomes of similar length (Figs 3A and 4). While we cannot entirely rule out residual uncertainty at the terminal regions of X, prior cytogenetic and genomic studies in sharks generally report short-to-intermediate X chromosomes, rendering a large error in X-chromosome length unlikely [12, 13]. This pattern persisted even when we examined only telomere-proximal genes, indicating that the reduction in synonymous substitution rates on the X chromosome is not due to gene positioning but rather reflects its chromosome-wide characteristic (Figs 3G and 4B). This is consistent with the male-driven evolution hypothesis: the mutation rate is higher in the male germline, and X chromosome spends proportionally less time in males, so neutral substitutions accumulate more slowly on the X chromosome [19–21]. Conversely, we found no clear evidence for Faster-X evolution, suggesting that adaptive evolution is not particularly accelerated at least in protein-coding genes. One potential explanation is the absence or incompleteness of dosage compensation in sharks. It has been proposed that the efficacy of Faster-X evolution may depend on the degree of dosage compensation [44, 45], and sharks have recently been reported to lack complete dosage compensation [13, 14], which could attenuate the signal of positive selection on the X chromosome. In the case of birds (ZZ/ZW), dosage compensation is generally incomplete on the Z chromosome, and many avian clades show faster-Z patterns often attributed to reduced effective population size with contributions from positive selection and male-biased mutation can elevate neutral rates on Z [46, 47]. Taken together, male mutation bias, hemizygosity, and dosage-compensation regimes determine how sex-chromosome signals vary among lineages.

Our results clarify that chromosome-length effects on substitution rates reflect a genome-wide, position-dependent “chromocline.” We infer that recombination rate heterogeneity underlies much of the cline, though direct recombination maps in the whale shark will be required to test this. In parallel with this recombination rate view, interpreting the X-specific slowdown requires resolving its counterpart, the Y chromosome. As in other sharks, the Y chromosome of the whale shark appears gene-poor and repeat-rich, rendering sequence recovery challenging. However, in other words, the gene-poor Y chromosome, together with the overall slow substitution rate and a karyotype with remarkably chromosome length variations, is a distinctive hallmark of sharks. These features position sharks as a powerful model to understand how genomic position, in concert with sex linkage, shapes the tempo and mode of gene evolution.

## Materials and methods

### DNA preparation

Ultrahigh molecular weight DNA was extracted from fresh blood sampled from a male adult whale shark (total length, 8.8 m at the time of sampling; individual ID, sRhiTyp1) at Okinawa Churaumi Aquarium originally for the previous study [9]. After storage at 4°C, the integrity of an aliquot of the extracted DNA was ensured to be high with pulse-field gel electrophoresis, and the DNA aliquot was subjected to whole-genome sequencing.

### Genome sequencing and assembly

The DNA extracted as described above was subjected to library preparation using the Ultra-Long DNA Sequencing Kit (Oxford Nanopore Technologies, SQK-ULK001). The prepared library was sequenced with R9.4.1 (FLO-PRO002) on PromethION. The raw sequencing output was processed with a basecaller Guppy Ver. 5 or 6 with super-accurate basecalling mode.

Long reads were assembled using Flye v2.9, Racon v1.4.22, and Medaka v1.4.3 [48, 49], resulting in 3,350 contigs with an N50 length of 17,247,868 bases. The contigs were then polished with the short-read data using FMLRC2 [50]. Hi-C reads from Yamaguchi et al. [9] (SRX15207325 and SRX15207324) were mapped to the polished contigs with HiC-Pro v3.1.0 with the default parameter [51], after building an index with Bowtie2 v2.4.4 and SAMtools v1.12 [52, 53]. Scaffolding was performed using YaHS v1.2 with the -r 2000,5000,10000,20000,50000, 100000,200000,500000,1000000,2000000,5000000,10000000,20000000,50000000,100000000,200000000,500000000 [54]. We manually curated the contact map using Juicebox v2.17.00 [55–57] (Fig. 1B). To visualize the contact map, we extracted KR-normalized Hi-C contact counts at 2.5 Mb resolution from the .hic file using the original assembly coordinate system with JuicerTools v1.9.9 [58], and visualized the matrix according to the manually curated assembly using a custom script [59].

To ensure consistency in sequence orientation with the previous genome assembly version (sRhiTyp1.1), we aligned the direction of the sequences against the former version using minimap2 v2.24 with the -x asm5 and –secondary=no options [60]. Based on the resulting PAF file, the orientation of each scaffold was determined by identifying the alignment strand with the highest total alignment length to a single reference scaffold. When no unique best alignment was available, the orientation was maintained.

To compare the new and old assemblies, we aligned our new assembly to the former version (sRhiTyp1.1) as a reference using minimap2 with the -x asm5 option [60]. Based on the alignment results, dot plots were generated using a customized version of the dotPlotly script with the options -m 1000 -q 1000 -s -l -x (Fig. 2B) [59, 61].

The assembly was evaluated using gVolante v2.0.0 and BUSCO v5.7.0 with the vertebrata_odb10 database (Fig. 1C and Supplementary Table S1) [62, 63].

### Gene prediction

We constructed a repeat model for each strain using RepeatModeler v2.0.5 with the -LTRStruct option and identified repeat sequences using RepeatMasker v4.1.5 with the options -nolow -xsmall [64, 65]. Next, we first performed training for Augustus using BUSCO v5.7.0_cv1 with the -augustus and -long options based on the vertebrata_odb10 dataset [63]. We then predicted the protein-coding exons using Augustus v3.2.3 with the options –softmasking=1 –alternatives-from-evidence=true and our own config file, incorporating 3 hint files derived from transcript evidence, homolog protein evidence, and repetitive regions [59, 66, 67]. To generate the transcript-based hint file, we performed adapter trimming to raw reads from RNA-seq using fastp [68] and mapped the trimmed reads to the assembly using HISAT2 v2.2.1 [69] (accession numbers are shown in Supplementary Table S2), which were previously reported by Yamaguchi et al. [9]. For a protein hint file, we mapped peptide data from the zebra shark (*S. tigrinum*, GCF_030684315.1 [70]) to the assembly using Exonerate v2.4.0 [71]. For a hint file from repeat regions, we used the RepeatMasker output. These outputs were converted to the appropriate format as instructed by the developer of Augustus [72].

### Identification of sex chromosomes

Short reads from female and male genomes were mapped to the scaffolds using BWA2 v2.2.1 with default parameters [73] (SRR19140286, SRR19140287, SRR19140281, and SRR19140282), which were previously reported by Yamaguchi et al. [9]. The read depth was calculated with SAMtools v1.19 and BEDtools v2.31.1 [53, 74], then normalized by the mean depth across the entire genome. We compared the female and male read depths and classified scaffolds with a female-to-male depth ratio of ~2 as putative X chromosome (Fig. 2A, C). One such chromosome, scaffold 40, was confirmed to be partially identical to a previously reported X chromosome [9] (Fig. 2B). To identify potential Y scaffolds, we searched for scaffolds meeting the criteria of a female-to-male depth ratio < 0.1, a female depth < 0.1, a male depth > 0.3, and a length > 100 Kbp. Based on these thresholds, we identified 2 scaffolds, scaffold 112 (645 Kbp), and scaffold 679 (195 Kbp) (Fig. 2D). We then examined the tandem repeats in these putative Y scaffolds using ModDotPlot v0.8.7, with each scaffold analyzed in an independent run [75] (Fig. 2E, F).

### Calculation of synonymous and non-synonymous substitution rate

We constructed ortholog groups (OGs) from the peptide sequences of whale shark (*R. typus*, this study), zebra shark (*S. tigrinum*, GCF_030684315.1 [70]), whitespotted bamboo shark (*Chiloscyllium plagiosum*, GCF_004010195.1 [76]), thorny skate (*Amblyraja radiata*, GCF_010909765.2 [77]), and elephant fish (*Callorhinchus milii*, GCF_018977255.1 [78]) using SonicParanoid v2.0.4 with default parameters [79], after selecting the longest isoform per gene using the agat_sp_keep_longest_isoform.pl script from AGAT v1.0.0 [80]. We then inferred phylogenetic trees for each OG using IQ-TREE v2.3.6 with the default parameters, after aligning the corresponding nucleotide sequences based on peptide alignments with MAFFT v7.526 and tranalign program of EMBOSS v6.6.0.0 [81–83]. Based on these phylogenetic trees, we used ETE3 v3.1.2 to identify OGs in which *R. typus* and *S. tigrinum* formed a monophyletic clade containing single-copy genes, resulting in 11,556 such OGs [84]. Next, for each OG, we calculated the *d_S_* and *d_N_* between these 2 species based on the YN model using the codeml program in PAML v4.9 with the following options: runmode = –2, model = 2, fix_kappa = 0, kappa = 2, fix_omega = 0, and omega = 1 [85]. We excluded the genes with *T* < 0.01 or *T* > 2. When we compared *d_S_* (or *d_N_*) between genes on autosomes and the X chromosome, we only utilized genes that were located on autosomes in both species (or on the X chromosome in both species), excluding 4 genes that had translocated between autosomes and the X chromosome.

## Availability of source code and requirements

Project name: ykawaguchi-jinta

Project homepage: https://github.com/YawakoK/ykawaguchi-jinta

License: MIT license

Operating system: Linux

Programming language: Python (v.3.6.8), R (v.4.3.2)

Package management: Python libraries: pandas (v.1.1.5), numpy (v.1.19.0), matplotlib (v.3.3.3); R packages: optparse (v.1.7.4), ggplot2 (v.3.4.4), plotly (v.4.10.4).

## Additional files

**Supplementary Table S1**. Statistics of 3 versions of whale shark genomes.

**Supplementary Table S2**. Accession number list of transcriptome data used for gene annotation.

**Supplementary Figure S1**. *d_S_* distribution within chromosomes 1–20 with quadratic regression results. Dots represent individual genes. Purple line and area show quadratic regression result and 95% confidence, respectively.

**Supplementary Figure S2**. *d_S_* distribution within chromosomes 1–20 with LOESS regression results. Dots represent individual genes. Orange line and area are LOESS regression result and 95% confidence, respectively.

## Supplementary Material

giag014_Supplemental_File

giag014_Authors_Response_To_Reviewer_Comments_original_submission

giag014_GIGA-D-25-00422_original_submission

giag014_GIGA-D-25-00422_Revision_1

giag014_Reviewer_1_Report_original_submission Reviewer 1 -- 11/28/2025

giag014_Reviewer_1_Report_Revision_1 Reviewer 1 -- 1/26/2026

giag014_Reviewer_2_Report_original_submission Reviewer 2 -- 12/15/2025

## Data Availability

The assembled sequence and nanopore raw reads have been deposited in the NCBI under BioProject PRJNA703743 (GenBank: GCA_021869965.2) and BioProject PRJNA1310834 (Accession: SRX30272874, SRX30272875, and SRX30272876), respectively. Previously published DNA short-read, Hi-C, and RNA-seq data used in this study are available under BioProjects PRJNA703743. Pre-processing inputs (prior to NCBI submission) and gene models are deposited on Figshare [86]. All custom scripts used in this study are deposited on GitHub [59]. All additional supporting data are available in the *GigaScience* repository, GigaDB [87].
