# Supplementary material for: Improved genome assembly of whale shark, the world’s biggest fish: revealing intragenomic heterogeneity in molecular evolution
Source: Gigascience. 2026 Feb 6;15:giag014. doi: 10.1093/gigascience/giag014 (PMC13100897; doi:10.1093/gigascience/giag014)
Supplement: giag014_Supplemental_File [file giag014_supplemental_file.pdf]

Supplementary Material for *GigaScience*

Improved genome assembly of whale shark, the world's biggest fish: revealing intragenomic heterogeneity in molecular evolution

Yawako W. Kawaguchi[1*], Rui Matsumoto[2,3], Shigehiro Kuraku[1,4,5*]

1. Molecular Life History Laboratory, Department of Genomics and Evolutionary Biology, National Institute of Genetics, Mishima, Shizuoka, 411-8540, Japan
2. Okinawa Churashima Research Center, Okinawa Churashima Foundation, Okinawa, 905-0206, Japan
3. Okinawa Churaumi Aquarium, Okinawa, 905-0206, Japan
4. Department of Genetics, Sokendai (Graduate University for Advanced Studies), Mishima, Shizuoka, 411-8540, Japan
5. Laboratory for Phyloinformatics, RIKEN Center for Biosystems Dynamics Research, Kobe, Hyogo, 657-0024, Japan

*Corresponding authors

Table S1 Statistics of three versions of whale shark genomes.

	RhiTyp_1.0	sRhiTyp1.1	sRhiTyp1.2
Assembly size [Mbp]	2,821	2,881	3,215
Number of scaffolds	136,451	16,776	3,201
N50 [Kbp]	3,127	70,797	65,379
L50	238	14	16
Length of 51st scaffold [Kbp]	-	2,449	3,965
Total length to 51st scaffold [Mbp]	-	2640	2800
Number of scaffolds > 1Mbp	690	51	67
Number of scaffolds > 10Mbp	24	46	48
Length of X chromosome [Kbp]	-	12,227	21,459
Number of gaps (>=5 N's)	56,773	95,223	470
BUSCO results for genomes	C:96.4%[S:94.4%,D:2.0%], F:2.1%,M:1.5%,n:3354,E:3.3%	C:84.2%[S:82.4%,D:1.8%], F:10.6%,M:5.2%,n:3354,E:4.2%	C:97.9%[S:95.8%,D:2.1%], F:0.9%,M:1.2%,n:3354,E:6.0%
BUSCO results for gene models*	-	C:82.6%[S:79.8%,D:2.8%], F:11.7%,M:5.7%,n:3354	C:89.0%[S:75.9%,D:13.1%], F:5.9%,M:5.1%,n:3354

* We used an NCBI RefSeq annotation as a gene model for sRhiTyp1.1 and our own annotation as one for sRhiTyp1.2

Table S2 Accession number list of transcriptome data used for gene annotation.

Accession number	Type	Sample
DRR111712	RNA-seq data, Blood cells	SAMD00098924
DRR111713	RNA-seq data, Blood cells	SAMD00098925
DRR111714	RNA-seq data, Blood cells	SAMD00098926
DRR182510	RNA-seq data, Blood cells	SAMD00175451
DRR182511	RNA-seq data, Blood cells	SAMD00175452
SRR19140227	RNA-seq data, Pancreas	SAMN28157481
SRR19140228	RNA-seq data, Intestine upper	SAMN28157480
SRR19140229	RNA-seq data, Spleen	SAMN28157479
SRR19140239	RNA-seq data, Kidney	SAMN28157478
SRR19140250	RNA-seq data, Liver	SAMN28157477
SRR19140261	RNA-seq data, Pituitary	SAMN28157476
SRR19140272	RNA-seq data, Intestine	SAMN28157475
SRR19140283	RNA-seq data, Stomach	SAMN28157474
SRR19140289	RNA-seq data, Uterus posterior	SAMN28157486
SRR19140290	RNA-seq data, Uterus middle	SAMN28157485
SRR19140291	RNA-seq data, Uterus anterior	SAMN28157484
SRR19140292	RNA-seq data, Follicle-large	SAMN28157483
SRR19140293	RNA-seq data, Follicle	SAMN28157482
SRR19140294	RNA-seq data, Eye	SAMN28157473
SRR19140295	RNA-seq data, Gill	SAMN28157472

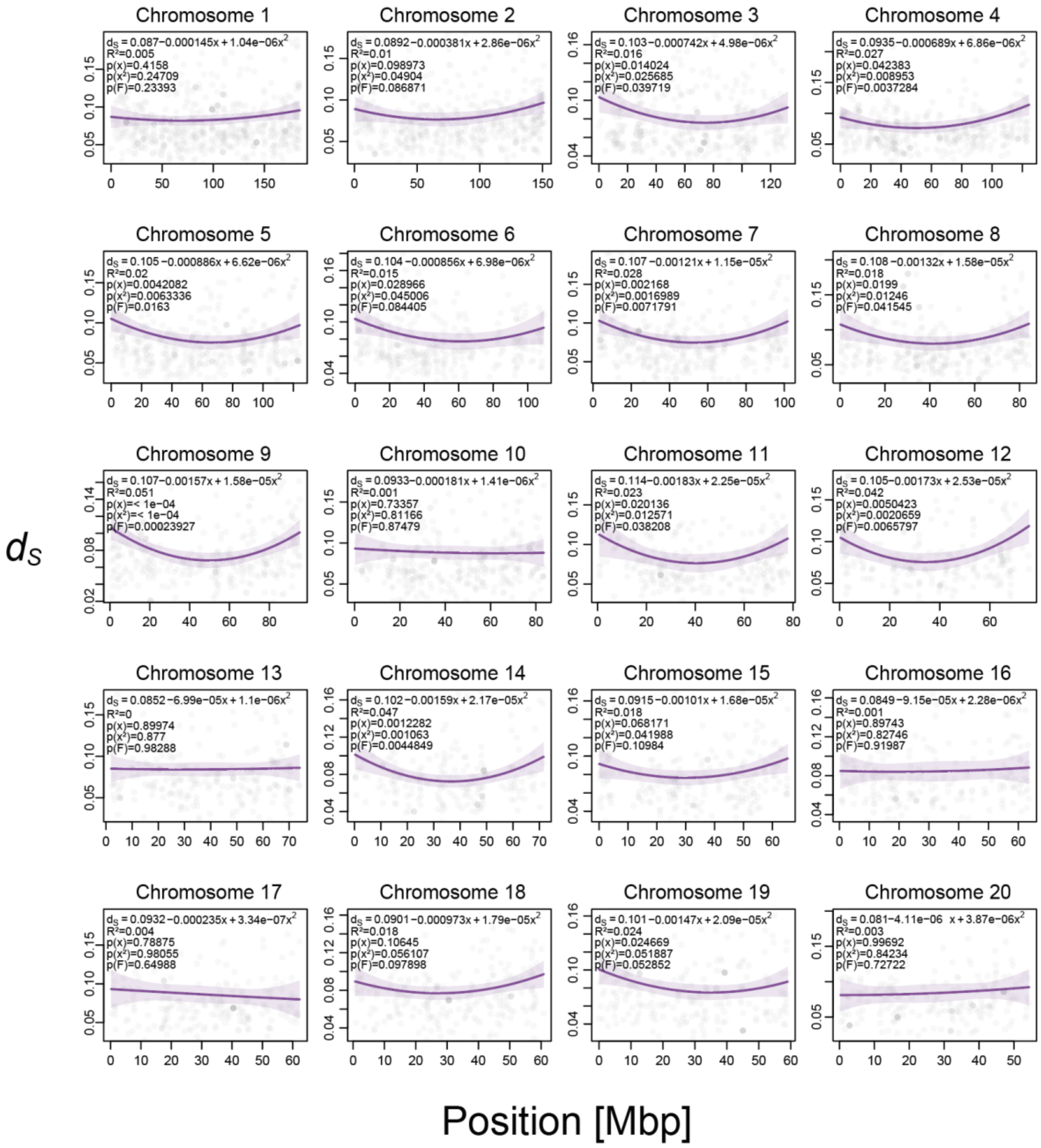

Figure S1. d_S distribution within chromosomes 1 to 20 with quadratic regression results.

Dots represent individual genes. Purple line and area show quadratic regression result and 95% confidence, respectively.

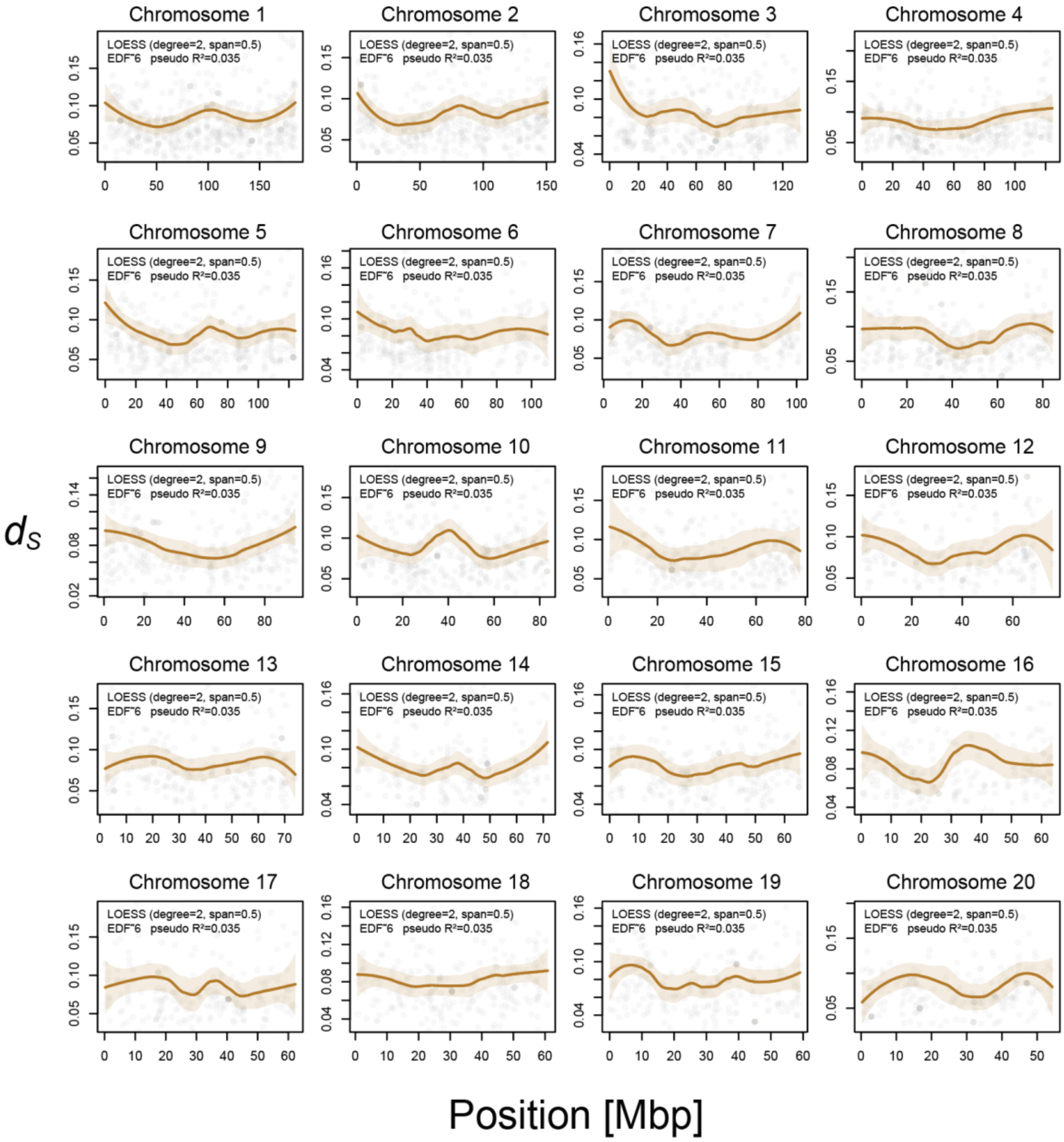

Figure S2. d_S distribution within chromosomes 1 to 20 with LOESS regression results. Dots represent individual genes. Orange line and area are LOESS regression result and 95% confidence, respectively.
