# Supplementary material for: Improved genome assembly of whale shark, the world’s biggest fish: revealing intragenomic heterogeneity in molecular evolution
Source: Gigascience. 2026 Feb 6;15:giag014. doi: 10.1093/gigascience/giag014 (PMC13100897; doi:10.1093/gigascience/giag014)
Supplement: giag014_Authors_Response_To_Reviewer_Comments_original_submission [file giag014_authors_response_to_reviewer_comments_original_submission.pdf]

Author's Response To Reviewer Comments

8 January 2026

GigaScience
Editor
Dear Yannan Fan,

We are grateful to the editors and the reviewer for their time and constructive comments to our manuscript, "Improved genome assembly of whale shark, the world's biggest fish: revealing intragenomic heterogeneity in molecular evolution" (GIGA-D-25-00422). Below, we respond to each comment from you and the reviewer (in italics) with references to the line numbers in the MainText_Revision_WithHighlightedChanges.pdf.
We hope that these revisions are sufficient to make our manuscript suitable for publication in Giga Science and look forward to hearing from you.

Sincerely,
Dr. Yawako W Kawaguchi

Response to the Editor
Your manuscript "Improved genome assembly of whale shark, the world's biggest fish: revealing "chromocline" in intragenomic heterogeneity" (GIGA-D-25-00422) has been assessed by our reviewers. Based on these reports, and my own assessment as Editor, I am pleased to inform you that it is potentially acceptable for publication in GigaScience, once you have carried out some essential revisions suggested by our reviewers.
Response: Thank you for handling our manuscript. Following your comments and those of the two reviewers, we have revised the manuscript text, figures, and the associated GitHub repository. The major revisions include (i) reducing the emphasis on the novelty of the term chromocline and clarifying its conceptual positioning, and (ii) revising the description of animal tissue sampling. Specifically, because all biological samples were obtained in the previous study (Yamaguchi et al. 2023), we have shortened the description of the original biological sampling process while retaining full sample traceability and provenance information. Detailed responses to all comments are provided below.

In addition, please register any new software application in the bio.tools and SciCrunch.org databases to receive RRID (Research Resource Identification Initiative ID) and biotoolsID identifiers, and include these in your manuscript. Computational workflows should be registered in workflowhub.eu and the DOIs cited in the relevant places in the manuscript. These will facilitate tracking, reproducibility and re-use of your tool.
Response: The scripts used in this study are auxiliary, project-specific analysis scripts developed solely to support the analyses presented in this manuscript. They are not standalone software applications or reusable computational workflows intended for general use. Therefore, we think a registration in bio.tools, SciCrunch (RRID), or WorkflowHub is not applicable. All scripts have been made publicly available in a GitHub repository and are described in the Methods section.

All web links and URLs should be given a reference number and included in the reference list rather than within the text of the manuscript. Please remove the URLs, cite them as reference and adjust the order of the reference accordingly. Except for "Abstract" and "Availability of Source Code and Requirements"
Response: We have removed all of the URLs in the main text and cited them in the reference list.

ORCIDs: Please update the authors' ORCID in the author list session.
Response: We have checked all ORCIDs are correct, but a "fetch" button in the "Authors" section can't work while adding Rui Matsumoto's ID (https://orcid.org/0000-0003-4057-8141). Please check and let us know what we can do.

Response to Reviewer #1
This is a solid piece of work. The improved Rhincodon typus assembly is genuinely better than previous versions—substantially higher contiguity, more complete BUSCO recovery, and a more convincing reconstruction of the X chromosome. The identification of candidate Y-linked scaffolds is plausible and methodologically defensible. The downstream evolutionary analyses are generally well executed, and the "chromocline" concept is interesting and—if interpreted with caution—potentially valuable for the field.
Response: Thank you very much for your positive and encouraging comments on our manuscript. We have carefully considered the reviewer's note regarding cautious interpretation of the "chromocline" concept and have revised the manuscript accordingly, as detailed below.

I really could not find any important flaws in the methodology. It would be interesting to discuss the small Y scaffolds with recently identified "Y" contigs. Apart from bamboo sharks, a recent assembly of the Carcharhinus amblyrhynchos genome also identified Y scaffolds and pseudo-autosomal regions on X.
Response: Thank you for your suggestion. We have added the mention of the Carcharhinus amblyrhynchos genome report as a reference to a recent report of shark sex chromosomes (L73, L75).

Furthermore, as the authors themselves not, several other papers (cited in text) have demonstrated similar "chromoclines". I would perhaps argue that the novelty here is slightly overstated, and defining this as "chromocline" risks sounding like terminology inflation rather than conceptual novelty.
Response: We thank the reviewer for this important and constructive comment. We agree that positional gradients in substitution rates along chromosomes were reported previously, including in several of the studies cited in our manuscript (e.g. in Xenopus and other vertebrates). Our intention was not to claim our discovery of a previously unknown phenomenon.
We have therefore revised the text to clarify that the novelty of our study lies in (i) demonstrating that chromosome-length effects on substitution rates are largely explained by intrachromosomal positional heterogeneity, and (ii) providing a unifying conceptual framework that links inter- and intra-chromosomal rate variation through distance from chromosome ends.
In line with this clarification, we have toned down our use of the term "chromocline" to emphasize that it is intended as a descriptive shorthand for a recurrent positional gradient, rather than as a claim of conceptual novelty. Accordingly, we have removed the term from the title, expanded the discussion of relevant prior work (particularly studies on subtelomeric regions) and softened language in the abstract and main text that could be interpreted as overstating novelty (L2-3, L29, L317-338).

Response to Reviewer #2
Overall, I find the manuscript well written and clear, and I think that the improved assembly it presents is a sufficiently large step forward to warrant publication. I have no issues with either the construction of the assembly, the procedure is state-of-the-art for non-model organisms, or the bioinformatic analysis regarding substitution rate variation across the chromosomes, which I find well done. Likewise, the detection of Y-chromosome fragments is convincing, in my opinion. In summary, I see no reason why this MS should not be accepted.
Response: Thank you for your positive assessment of our manuscript. We are really glad to hear that.
Minor comment:
If possible, improve the quality of figure panels 1B (in particular the scale bar) and 2F.
Response: We have updated Figures 1B and 2F, and added descriptions for the figures in the main manuscript (L424-427, L470-471) and an own custom script in GitHub (https://github.com/YawakoK/ykawaguchi-jinta).

Editorial Manager®

Cookie Preference Center

We use cookies which are necessary to make our site work. We may also use additional cookies to analyze, improve and personalize our content and your digital experience. For more information, see our Cookie Policy.

You may choose not to allow some types of cookies. However, blocking some types may impact your experience of our site and the services we are able to offer. See the different category headings below to find out more or change your settings.

Allow all

Manage Consent Preferences

Strictly Necessary Cookies

Always active

These cookies are necessary for the website to function and cannot be switched off in our systems. They are usually only set in response to actions made by you which amount to a request for services, such as setting your privacy preferences, logging in or filling in forms. You can set your browser to block or alert you about these cookies, but some parts of the site will not then work.

Functional Cookies

These cookies enable the website to provide enhanced functionality and personalisation. They may be set by us or by third party providers whose services we have added to our pages. If you do not allow these cookies then some or all of these services may not function properly.

Performance Cookies

These cookies allow us to count visits and traffic sources so we can measure and improve the performance of our site. They help us to know which pages are the most and least popular and see how visitors move around the site.

Targeting Cookies

These cookies may be set through our site by our advertising partners. They may be used by those companies to build a profile of your interests and show you relevant adverts on other sites. If you do not allow these cookies, you will experience less targeted advertising.

Cookie List

Clear

Apply Cancel

Consent Leg.Interest

Confirm my choices

Powered by onetrus
